# Fatigue in patients with cancer receiving outpatient chemotherapy: a prospective two-center study

**DOI:** 10.1186/s40780-023-00275-0

**Published:** 2023-02-17

**Authors:** Takuya Fujihara, Motohiko Sano, Yutaka Negoro, Shinji Yamashita, Hideya Kokubun, Ryoichi Yano

**Affiliations:** 1 Department of Pharmacy Services, Saitama Medical Center, 1981 Kamoda, Kawagoe, Saitama 350-8550 Japan; 2 Division of Applied Pharmaceutical Education and Research, Hoshi University, 2-4-41 Ebara, Shinagawa-Ku, Tokyo, 142-8501 Japan; 3 Department of Pharmacy, University of Fukui Hospital, 23-3 Matsuoka Shimoaizuki, Eiheiji, Fukui 910-1193 Japan; 4 Center for Experiential Pharmacy Practice, Tokyo University of Pharmacy and Life Sciences, 1432-1, Horinouchi, Hachioji, Tokyo, 192-0392 Japan; 5 Education and Research Center for Clinical Pharmacy, Faculty of Pharmacy, Osaka Medical and Pharmaceutical University, 4-20-1 Nasahara, Takatsuki, Osaka 569-1094 Japan

**Keywords:** Cancer, Cancer-related fatigue, ESAS-r-J, Outpatient, Tiredness

## Abstract

**Background:**

Cancer-related fatigue (CRF) is one of the most common symptoms in patients with cancer. However, CRF has not been sufficiently evaluated as it involves various factors. In this study, we evaluated fatigue in patients with cancer receiving chemotherapy in an outpatient setting.

**Methods:**

Patients with cancer receiving chemotherapy at the outpatient treatment center of Fukui University Hospital and Saitama Medical University Medical Center Outpatient Chemotherapy Center were included. The survey period was from March 2020 to June 2020. The frequency of occurrence, time, degree, and related factors were examined. All patients were asked to fill out the Edmonton Symptom Assessment System Revised Japanese version (ESAS-r-J) questionnaire, which is a self-administered rating scale, and patients with ESAS-r-J “Tiredness” scores of ≥ 3 were evaluated for factors related to tiredness, such as age, sex, weight, and laboratory parameters.

**Results:**

A total of 608 patients were enrolled in this study. Fatigue after chemotherapy occurred in 71.0% of patients. ESAS-r-J “Tiredness” scores of ≥ 3 were observed in 20.4% of patients. The factors related to CRF were low hemoglobin level and high C-reactive protein level.

**Conclusions:**

Twenty percent of patients receiving cancer chemotherapy on an outpatient basis had moderate or severe CRF. Patients with anemia and inflammation are at increased risk of developing fatigue after cancer chemotherapy.

## Background

Cancer-related fatigue (CRF) [1] is a common complication in patients with cancer [2], and it cannot improve easily with rest and sleep. CRF occurs before the initiation of treatment for cancer, such as radiotherapy and chemotherapy, increases during treatment [3, 4], and persists after treatment completion; thus, it greatly impairs patients’ quality of life. Bower et al. [5] indicated that approximately 25–30% of patients with cancer who have completed treatment have persistent fatigue for several years. In addition, Groenvold et al. reported that increased fatigue during treatment reduces overall survival, indicating that fatigue is a key issue that should be addressed when determining cancer treatment plans [6]. However, fatigue is difficult to assess because it is largely subjective; therefore, there are very few studies on CRF. Further, no standard treatment for fatigue has been established yet. The 2020 European Society of Medical Oncology clinical practice guidelines for CRF recommend short-term steroidal treatment and exercise, but there is insufficient evidence for their efficacy. Some reports have shown that methylphenidate is effective, but no guidelines currently recommend its use [7].

CRF is broadly classified into primary CRF, which is primarily caused by tumors or cancer treatments, and secondary CRF, which is caused by comorbidities that frequently occur in patients with cancer [8]. However, it is difficult to clarify the etiology of CRF because various factors are involved [9]. Therefore, it is necessary to carefully evaluate the etiology of fatigue in individual patients. Although some studies on CRF have been conducted in the last 20 years, and the National Comprehensive Cancer Network has developed CRF guidelines in 2000 [10], most previous studies have evaluated CRF only in conjunction with fatigue associated with carcinomas. Furthermore, these studies were not conducted in Asian patients. To address these issues, we investigated the occurrence of fatigue in patients with cancer receiving chemotherapy at two outpatient facilities in Japan and examined the frequency and degree of CRF, in addition to correlated factors.

## Patients and methods

### Design and participants

This prospective study included patients who received chemotherapy at the outpatient treatment center of Fukui University Hospital and Saitama Medical University Medical Center Outpatient Chemotherapy Center. The survey period was from March 2020 to June 2020. The selection criteria were outpatient treatment including undergoing curative treatment or palliative treatment for pathologically diagnosed cancer, Eastern Cooperative Oncology Group Performance Status (PS) of 0–3, ability to understand the Japanese language, and age between 20 and 85 years. Patients with a severe psychiatric disorder or cognitive impairment that interferes with daily living, as diagnosed by a physician, were excluded.

### Survey method

**Fig. 1:**
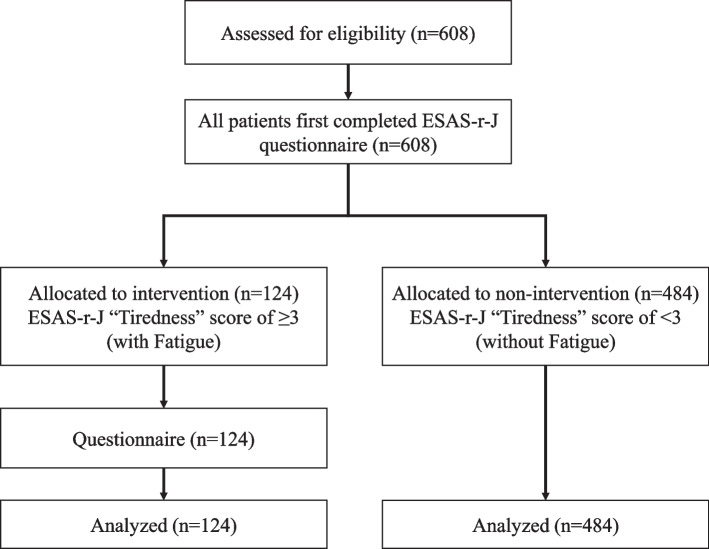
A flow chart of the study

A flow chart of the study is presented in Fig. 1. All patients first completed the Edmonton Symptom Assessment System Revised Japanese version (ESAS-r-J) questionnaire [11] In addition, nformation on age, sex, body weight within the last month, cancer type, and laboratory parameters (hemoglobin [Hb], C-reactive protein [CRP], serum sodium, serum potassium, serum calcium, blood urea nitrogen, serum creatinine, and serum albumin levels) were extracted from the electronic medical records of the patients to investigate their backgrounds. Patients with ESAS-r-J score of ≥ 3 were interviewed by the medical professionals using the questionnaire (onset timing of fatigue, PS ≥ 2 or < 2, disease complications, sleep disorders, dietary intake, edema, diarrhea, self-treatment for fatigue).

### Assessment of factors associated with fatigue

The ESAS-r-J questionnaire was used to assess symptoms (pain, tiredness, drowsiness, nausea, lack of appetite, shortness of breath, depression, anxiety, others). The ESAS-r-J scoring system rates the intensity of each symptom on an 11-point scale from 0 (no symptom) to 10 (worst possible symptom). Factorial analyses of fatigue were performed by comparing patients with an ESAS-r-J score of ≥ 3 for “Tiredness” with those with an ESAS-r-J score of < 3.

### Statistical analysis

Statistical calculation of the planned number of enrollees was not performed, and the upper limit was not set. Further, statistical analysis was performed on the number of samples excluding each factor missing value. The patients were classified into two groups based on their ESAS-r-J score for “Tiredness.” Fisher’s exact test was used to compare nominal variables, the Shapiro–Wilk test was used to confirm normality, and the Wilcoxon rank sum test or unpaired t-test was used to compare continuous variables. In addition, multiple logistic regression analysis was performed in patients with a “Tiredness” score of ≥ 3. The multicollinearity of the independent variables was confirmed in advance using scatter plots. A *p*-value less than 0.05 was considered statistically significant. Statistical analysis was performed using JMP Pro statistical software (version 13.0; SAS Institute Inc., Cary, NC).

### Ethical considerations

This study was approved by the Research Ethics Review Committee for Medical Research of Fukui University (Reference No.: 20190092) and the Ethics Committee of the Center for Medical Science of Saitama University (Application No.: 2277). Information was obtained from the participants in this study through interviews and questionnaires. Since no invasive procedures or interventions were performed and no samples were obtained from patients, the requirement for written informed consent was waived. In addition, the details of the study were posted at an easily accessible location in the treatment room where chemotherapy was administered. Written information on the study and a consent form were provided to each patient who was interviewed (those with an ESAS-r-J “Tiredness” score of ≥ 3), and their willingness to participate in the study was verbally confirmed. Personal information was anonymized using case numbers, and a table for correspondence between case numbers and patient identification information (e.g., medical record numbers) was prepared and managed.

## Results

### Patient characteristics

A total of 608 patients with cancer (53.5% men, 46.5% women; mean age, 66.3 ± 11.8 years) were included in the study (Table 1). The patients had slightly low Hb and albumin levels and slightly high CRP levels, but other laboratory parameters remained within the normal range. In terms of cancer site, 16.9% of patients had hematologic cancer, 16.4% had lung cancer, and 15.1% had colorectal cancer. Nevertheless, patients with a relatively wide range of cancer sites, including the breast and pancreas, were included in the study.

**Table 1:** Patient characteristics

	Percentage or mean ± SD (Number of patients)
Sex (male/female)	53.5%/46.5% (608)
Age (years)	66.3 ± 11.8 (608)
BW (kg)	57.4 ± 11.7 (603)
Laboratory parameter levels	
Hb (g/dL)	11.6 ± 1.8 (593)
CRP (mg/dL)	0.91 ± 1.68 (496)
Na (mmol/L)	141.3 ± 2.6 (568)
K (mmol/L)	4.2 ± 0.4 (568)
Corrected Ca (mg/dL)	9.3 ± 0.4 (419)
BUN (mg/dL)	15.7 ± 5.9 (572)
Scr (mg/dL)	0.84 ± 0.56 (580)
Alb (g/dL)	3.8 ± 0.5 (536)
Primary cancer site	
Hematologic	16.9% (103)
Lung	16.4% (100)
Colorectal	15.1% (92)
Breast	10.2% (62)
Gynecologic	9.4% (57)
Urinary	9.4% (57)
Pancreatic	7.6% (46)
Gastric	7.2% (44)
Head and neck	1.3% (8)
Others	6.4% (39)

Corrected Ca = Ca + 4—Alb (if Alb < 4)

*Abbreviations: SD* Standard Deviation, *BW* Body weight, *Hb* hemoglobin, *CRP* C-reactive protein, *Na* Sodium, *K* Potassium, *Ca* Calcium, *BUN* Blood urea nitrogen, *Scr* Serum creatinine, *Alb* Serum albumin

### ESAS-r-J scoring

Table 2 shows the ESAS-r-J scores of the participants. “Anxiety” (24.0%) was the most commonly observed variable for which patients were symptomatic (≥ 3), followed by “Tiredness.”

**Table 2:** Number of patients with each symptom based on the ESAS-r-J score (*n* = 608)

	0	1 or 2	≧3
	n (%)	n (%)	n (%)
Pain	404 (66.4)	131 (21.5)	72 (11.8)
Tiredness	310 (51.0)	174 (28.6)	124 (20.4)
Drowsiness	367 (60.4)	142 (23.4)	99 (16.3)
Nausea	516 (84.9)	65 (10.7)	26 (4.3)
Lack of appetite	420 (69.1)	108 (17.8)	79 (13.0)
Shortness of breath	427 (70.2)	109 (17.9)	72 (11.8)
Depression	364 (59.9)	138 (22.7)	106 (17.4)
Anxiety	317 (52.1)	145 (23.8)	146 (24.0)
Constipation	410 (67.4)	109 (17.9)	85 (14.0)
Diarrhea	496 (81.6)	71 (11.7)	41 (6.7)
Skin disorders	416 (68.4)	104 (17.1)	86 (14.1)
Neuropathy	448 (73.7)	80 (13.2)	80 (13.1)
Hair loss	408 (67.1)	108 (17.8)	92 (15.1)

*ESAS-r-J* Edmonton Symptom Assessment System Revised Japanese version

### Factors associated with fatigue

**Fig. 2:**
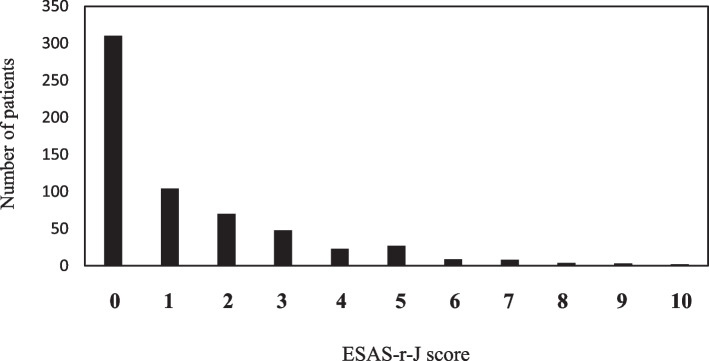
Distribution of patients by “Tiredness” score. ESAS-r-J, Edmonton Symptom Assessment System Revised Japanese version

Figure 2 shows the distribution of ESAS-r-J scores for “Tiredness.” Among patients with a “Tiredness” score ≥ 3, most patients with score 3, 4, or 5 had relatively mild symptoms. Table 3 shows the results of the interviews conducted by healthcare professionals in patients with “Tiredness” scores of ≥ 3 on the ESAS-r-J. Fatigue after chemotherapy occurred in 71.0% of patients, and 58.9% of patients had other comorbidities, such as hypertension and diabetes mellitus.

**Table 3:** Interview results in patients with fatigue (*n* = 124)

	n (%)
Onset of fatigue	
Before cancer chemotherapy	27 (21.8)
After cancer chemotherapy	88 (71.0)
Unknown	9 (7.3)
PS ≥ 2	46 (37.1)
With disease complication (HT, DM, etc.)	73 (58.9)
With sleep disorders	32 (25.8)
Reduced dietary intake	35 (28.2)
With edema	32 (25.8)
With diarrhea	19 (15.3)
Self-treatment for fatigue (acupuncture and moxibustion, aromatherapy, etc.)	19 (15.3)

*PS* Eastern Cooperative Oncology Group Performance Status, *HT* Hypertension, *DM* Diabetes

Table 4 shows the comparison of background factors between patients with a “Tiredness” score ≥ 3 and those with score < 3. The results of the multiple logistic regression analyses performed with factors having significant differences in Table 4 as independent variables are shown in Table 5. Multiple collinearities of the independent variables were confirmed using a priori scatter plots, and there were no variables with linear relationships. Using “Tiredness” scores as the dependent variable, the results of the model χ^2^ test were significant (*p* < 0.001). Hb (odds ratio [OR] 0.816, 95% confidence interval [CI] 0.675–0.983) and CRP (OR 1.355, 95% CI 1.123–1.673) levels were extracted as significant variables. The discriminant predictive value was relatively good (81.6%).

**Table 4:** Comparison of the patients’ background factors according to fatigue

	Without fatigue	With fatigue	*P*-value
	(Number of patients)		
Age (years, mean ± SD)	66.4 ± 11.4 (484)	66.1 ± 13.3 (124)	0.6187^c^
Sex (male/female, %)	54.5/45.5 (484)	49.2/50.8 (124)	0.3134^a^
BW (kg, mean ± SD)	58.0 ± 11.8 (479)	55.2 ± 11.0 (124)	0.0227^c^
Laboratory parameter levels (mean ± SD)			
Hb (g/dL)	11.8 ± 1.8 (472)	11.0 ± 1.9 (121)	< 0.0001^b^
CRP (mg/dL)	0.71 ± 1.36 (393)	1.68 ± 2.42 (103)	< 0.0001^c^
Na (mmol/L)	141.5 ± 2.5 (449)	140.5 ± 2.8 (119)	0.0001^c^
K (mmol/L)	4.2 ± 0.4 (450)	4.2 ± 0.4 (118)	0.5770^c^
Corrected Ca (mg/dL)	9.3 ± 0.4 (330)	9.4 ± 0.4 (89)	0.0343^c^
BUN (mg/dL)	15.7 ± 5.4 (454)	15.5 ± 7.4 (118)	0.3630^c^
Scr (mg/dL)	0.84 ± 0.52 (462)	0.84 ± 0.69 (118)	0.6055^c^
Alb (g/dL)	3.8 ± 0.4 (424)	3.6 ± 0.5 (112)	< 0.0001^c^

*Abbreviations: SD* Standard Deviation, *BW* Body weight, *Hb* Hemoglobin, *CRP* C-reactive protein, *Na* Sodium, *K* Potassium, *Ca* Calcium, *BUN* Blood urea nitrogen, *Scr* Serum creatinine, *Alb* Serum albumin

^a^Fisher’s exact test

^b^Student’s unpaired t-test

^c^Wilcoxon rank sum test

**Table 5:** Multiple logistic regression analysis in patients with fatigue

	*P*-value	Odds ratio	95% confidence interval
BW (kg, mean ± SD)	0.2547	1.500	0.746–3.015
Hb level (g/dL)	0.0325	0.816	0.675–0.983
CRP level (mg/dL)	0.0013	1.355	1.123–1.673
Na (mmol/L)	0.2302	0.933	1.045–1.071
Corrected Ca (mg/dL)	0.7909	1.120	0.483–2.606
Alb (g/dL)	0.5147	0.766	0.342–1.716

Model χ^2^ test: *p* < 0.001

Discriminative predictive value: 81.6%

*Abbreviations: BW* Body weight, *Hb* Hemoglobin, *CRP* C-reactive protein, *Na* Sodium, *Ca* Calcium, *Alb* Serum albumin

## Discussion

This study was performed to clarify the actual conditions associated with fatigue related to cancer treatment by simultaneously evaluating fatigue in patients with cancer receiving chemotherapy and studying the existing complications and treatment interventions. We defined “symptomatic” as an ESAS-r-J score of ≥ 3. Previous reports evaluating fatigue have followed the National Comprehensive Cancer Network guidelines [12] and considered ESAS scores ≥ 4 as indicative of symptomatic fatigue [13, 14]. We used a score of ≥ 3 because patients receiving treatment at outpatient clinics may have mild lethargic symptoms, which can be missed during their outpatient visits.

Of the 608 patients surveyed, 124 (20.4%) experienced fatigue, which was a higher rate than that reported in previous studies. Although comparing the results of different studies is difficult because of differences in timing and patient characteristics, all the symptoms observed in our study are among the top complaints of patients with cancer [15, 16]. However, in many reports, moderate to severe fatigue was observed in 30–60% of patients with cancer [17], which is slightly higher than what we observed. It has been observed that the prevalence rate of fatigue varies depending on the patient’s background, treatment received, and method of assessing fatigue [18]. Risk factors for fatigue include low PS, chemoradiation, female sex, insomnia, pain, and depression [19]. Therefore, the lower proportion of patients complaining of fatigue in our study compared with that in previous studies may be attributable to the relatively high PS of the patients. Depression, which was common among patients with fatigue in our study, has been identified as a risk factor for fatigue in several studies [20, 21], in consistent with our results. Nausea was less common than other symptoms among “symptomatic” patients, which may reflect recent advances in supportive care.

In addition to examining the prevalence of fatigue using the ESAS-r-J questionnaire, healthcare professionals interviewed the patients with fatigue regarding the time of appearance of fatigue, comorbidities, sleeping conditions, and dietary intake. Patients with “Tiredness” scores of ≥ 3 often developed fatigue after chemotherapy. Regarding the timing of fatigue, a meta-analysis of patients with rectal cancer before and after treatment has shown that fatigue peaks at 1 month after treatment initiation and then decreases [22]. This trend for fatigue after chemotherapy is similar to what we observed, in which more patients had fatigue after chemotherapy than before it. Further, the prevalence of fatigue in patients with comorbidities such as hypertension and diabetes mellitus was 58.9%. This confirms that fatigue in patients with cancer is multidimensional and that patients with hypertension, diabetes, and other comorbidities are more likely to develop fatigue than those without comorbidities. In addition, multivariate logistic regression analysis was performed, including age, sex, body weight, and laboratory parameters (Hb, CRP, sodium, potassium, calcium, blood urea nitrogen, creatinine, and albumin levels) as independent variables. The result indicated that low Hb and high CRP levels were significant independent predictors of fatigue. The ratio of CRP to Alb is an indicator of the degree of improvement in nutritional status and recovery from invasion. High CRP and low Alb levels are considered plausible factors associated with fatigue. Fatigue, anemia, and inflammation have been previously shown to be correlated in patients with cancer, suggesting that patients with baseline anemia and inflammation may be at increased risk of developing fatigue after cancer chemotherapy [23, 24]. With regard to fatigue after chemotherapy and the above two factors, the drugs used have also been investigated. Zhang et al. [25] reported that the administration of platinum-based drugs may induce inflammation and worsen anemia, leading to CRF. Therefore, it is necessary to investigate the improvement of anemia and interventions targeting inflammation.

There are some limitations to this study. First, this study was conducted at only two institutions. However, the sample size was 608, which is good, and we believe that we were able to include a large number of patients. Second, this study was conducted among outpatients in Japan. However, there are no reports of racial differences in fatigue following chemotherapy, and the results of this study may be useful because chemotherapy is being increasingly administered in an outpatient setting. Third, this study design did not allow us to obtain the presence of comorbidities, cancer type, treatment line, chemotherapy regimen, and PS data on complications in patients without fatigue. Future studies are warranted to compare these factors in patients with and without fatigue to appreciate their effects on CRF.

In conclusion, among patients who receive chemotherapy in an outpatient setting, those with anemia and high CRP level tend to develop fatigue. Therefore, it is important to provide adequate support to these patients. Once the risk factors for fatigue have been identified, nutritional therapy, anamorelin administration or exercise therapy may be advised to the patients to improve their symptoms.

## Data Availability

The datasets used and/or analyzed during the current study are available from the corresponding author on reasonable request.

## Abbreviations

Alb: Serum albumin
BUN: Blood urea nitrogen
BW: Body weight
CI: Confidence interval
CRF: Cancer-related fatigue
CRP: C-reactive protein
DM: Diabetes
ESAS-r-J: Edmonton Symptom Assessment System Revised Japanese version
Hb: Hemoglobin
HT: Hypertension
OR: Odds ratio
PS: Eastern Cooperative Oncology Group Performance Status
Scr: Serum creatinine
SD: Standard deviation
